# Comment on Sokawa, Y. Radiation-Induced Childhood Thyroid Cancer after the Fukushima Daiichi Nuclear Power Plant Accident. *Int. J. Environ. Res. Public Health* 2024, *21*, 1162

**DOI:** 10.3390/ijerph22050674

**Published:** 2025-04-25

**Authors:** Toshiko Kato, Mamoru Hayashi, Tadashi Hongyo

**Affiliations:** 1 Independent Researcher, Nara 630-8242, Japan; 2 Faculty of Human Development, University of Toyama, Toyama 930-8555, Japan; 3 Department of Radiation Biology, Health Sciences, Graduate School of Medicine, Osaka University, Osaka 565-0871, Japan; hongyo@sahs.med.osaka-u.ac.jp

Sokawa studied the radiation effect on thyroid cancer after the Fukushima Daiichi Nuclear Power Plant Accident [1]. We agree that radiation from the accident caused the development of thyroid cancer because there were clear regional differences in the incidence of childhood thyroid cancer, which agrees with our result of the area’s dose–response relationship in thyroid cancer incidence [2]. In Sokawa’s article “Radiation-Induced Childhood Thyroid Cancer after the Fukushima Daiichi Nuclear Power Plant Accident” [1], he assigned a considerable portion of childhood thyroid cancer cases detected in the Fukushima Health Management Survey (FHMS) to the “common case” not affected by radiation exposure.

According to Japan’s cancer statistics, the incidence rate of thyroid cancer in people aged 0–19 years at diagnosis increased the average annual incidence rate of 0.22/10^5^ PY in 2003–2010 by 2.6 times to 0.55/10^5^ PY after the accident (2011–2015) [3]. This increase suggested that the thyroid cancer incidence rate increased in Fukushima and other contaminated prefectures in eastern Japan due to radiation exposure from the nuclear accident.

Sokawa plotted the incidence rate observed in four areas—A, B, C, and D—based on the basic survey (BS) and the cumulative incidence rates from the BS and the first full-scale survey (FSS1) versus the time elapsed from the accident to the end of the survey in years (Figure 1 [1]). He estimated the incidence rate at the time of the accident using the intercept between the *y*-axis and the line extrapolated, connecting the points plotted for the BS and the cumulative points plotted for the BS + FSS1 in each area. Sokawa reported that the four lines intersected at almost the same point, at an average value of 21.9 ± 2.7/10^5^ examinees, and assumed that this value indicates the incidence rate of “common cases” not affected by radiation exposure at the time of the accident.

However, Sokawa adopted an incorrect timeline for the BS and FSS1 of area A. Although the BS started 7 months after the accident and the survey took about 2 years, he used a timeline of only 1 year from the accident to the end of the BS. He adopted a three-year inspection period from the BS to FSS1 for area A but used the same periods for all other areas, about 2 years. If the time elapsed from the accident to the end of the BS and FSS1 of area A are corrected based on the real timeline, 1.5 and 3.5 years (with an inspection period of 2 years), and 1.3 and 3.7 years (2.4 years), the intercept with the *y*-axis will decreases from 23.8/10^5^ examinees (Sokawa’s results) to −3.6 and 10.1/10^5^ examinees, respectively (Figure 1). Because the intercept of areas B~D was at 19.3–22.5/10^5^ examinees, the intercepts for all four areas at the time of the accident do not coincide. Therefore, the common intercept among the four areas was a special case for an incorrect timeline for the BS and FSS1 of area A.

We cannot estimate the incidence rate just before the accident by linearly extrapolating the cumulative incidence rates of BS and FSS1 as a function of the years elapsed since the accident, because the growth of thyroid cancer, stimulated by the second most intense radiation exposure after the Chernobyl nuclear accident, does not follow a steady pattern. The annual incidence rate in the Fukushima prefecture just before the nuclear accident was presumably close to the average incidence rate across Japan, 0.22/10^5^ PY. The incidence rate at the time of the accident of 21.9 ± 2.7/10^5^ estimated by Sokawa is considered to be a significant overestimation.

In addition to the above discussion, much evidence of the radiation effect on thyroid cancer in Fukushima has been reported. First, Shimura of FMU, at the 67th Japan Thyroid Association meeting in 2024, revealed that “the majority of detected thyroid cancer patients in the second TUE (83%) and third TUE (64%) had no nodules in the preceding examination” [4]. This indicates that thyroid cancer cases among residents who were severely exposed in Fukushima began to appear after the accident and exhibited extremely rapid growth, progressing from zero to diagnosis within two years. These characteristics of thyroid cancer detected in the FHMS are quite different from those of the slow-growing, naturally occurring thyroid cancers [5].

Second, Shibata et al. [6] and Demidchik et al. [7] found that thyroid cancers were detected among children born before the Chernobyl accident, whereas no cases were detected by ultrasound screening among children born after the accident. This demonstrates that there was no screening effect in detecting childhood thyroid cancer. These observations indicated that the excess thyroid cancer was not due to screening effects, and led to the international recognition of radiation-induced thyroid cancer in Chernobyl.

Sokawa is concerned that the detection methods used for the cancer statistics and in the FHMS are completely different, and it is not possible to directly compare the detection rates of the two. However, both detections are nearly the same because both are performed following the guidelines of the Japan Association of Breast and Thyroid Sonology. Even if a small nodule is detected by a highly sensitive ultrasound device, a cytology test will not be performed up to a 20 mm nodule if there is no suspicion of malignancy, and it will not lead to the detection of cancer. Highly sensitive ultrasound devices are effective in determining whether a growing nodule is suspicious for malignancy, and the possibility of overdiagnosis is reduced through the use of highly sensitive ultrasound.

The FHMS committee evaluated the number of thyroid cancer cases detected during the first and second TUEs (2011–2015) as being dozens of times higher than that estimated based on Japanese cancer statistics [5,8]. Tsugane reported the detection of dozens-fold excess thyroid cancer likely either due to an excess incidence caused by certain factors or the overdiagnosis of cancers that would not have been clinically diagnosed or that would not have led to death in the future [5]. The only possible explanation for the major trend in detected thyroid cancers in FHMS [4]—namely, a small nodule growing rapidly—is the effect of radiation exposure. Most cancer detections except naturally occurring thyroid cancer (about 1/60) are most probably be associated with radiation exposure if we consider that no screening effect was observed by ultra-sound among children born after the accident in Chernobyl.

The assumption in Sokawa’s paper that a considerable portion of childhood thyroid cancer cases are “common cases” unaffected by the nuclear accident was found to have no basis in his analysis and contradicted evidence of the extremely rapid growth of thyroid cancer observed after the accident. The conclusion of “the existence of considerable amount of common case of thyroid cancer not affected by radiation exposure” should be withdrawn.

Although “incidence” and “prevalence” seemed to have been confused for one another in Sokawa’s paper, we retained the use of “incidence” here.

Sokawa incorrectly indicated that the nine patients detected in the third FSS in area A and in the second FSS in area B are likely (>100%) not affected by radiation exposure (Figure 3 [1]). His paper inaccurately determined the effect of radiation exposure on thyroid cancer patients without evidence, which may cause suffering for young patients.

Therefore, the author should publish a corrigendum and stress “no existence of considerable amount of common case of thyroid cancer not affected by radiation exposure”. The annual incidence rate of naturally occurring thyroid cancer just before and after the accident in Fukushima residents should be considered to be between 0.22/10^5^ PY in 2003–2010 and 0.55/10^5^ PY in 2011–2015 of cancer statistics of Japan [3].

## Figures and Tables

**Figure 1:**
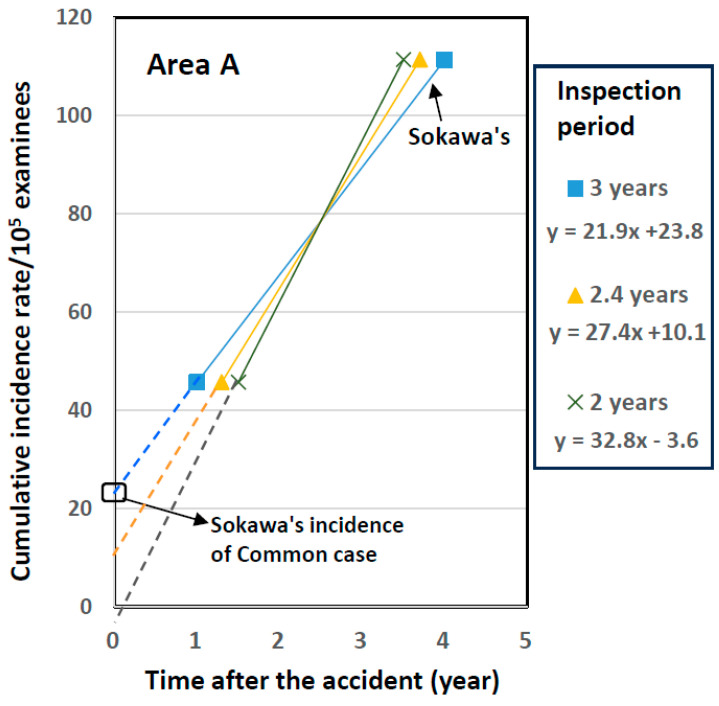
Cumulative incidence rate versus years after the nuclear accident for area A during inspection periods of 3, 2, and 2.4 years between the BS and FSS1.

## References

[1] Sokawa Y. (2024). Radiation-Induced Childhood Thyroid Cancer after the Fukushima Daiichi Nuclear Power Plant Accident. Int. J. Environ. Res. Public Health.

[2] Kato T., Yamada K., Hongyo T. (2023). Area Dose–Response and Radiation Origin of Childhood Thyroid Cancer in Fukushima Based on Thyroid Dose in UNSCEAR 2020/2021: High ^131^I Exposure Comparable to Chernobyl. Cancers.

[3] Cancer Statistics in Japan, National Cancer Registries in Japan (1975–2015), (2016–2018). https://ganjoho.jp/reg_stat/index.html.

[4] Shimura H. Current Status of Thyroid Examination and Findings on Thyroid Nodules and Cancer in Fukushima Health Management Survey. Proceedings of the 67th Annual Meeting of the Japan Thyroid Association.

[5] Tsugane S., Katanoda K. Estimation of the Number of Prevalent Thyroid Cancer Patients in Fukushima Prefecture in 2010. Thyroid Examination Evaluation Subcommittee 2014. https://www.pref.fukushima.lg.jp/uploaded/attachment/91000.pdf.

[6] Shibata Y., Yamashita S., Masyakin V.B., Panasyuk G.D., Nagataki S. (2001). 15 years after Chernobyl: New evidence of thyroid cancer. Lancet.

[7] Demidchik Y.E., Saenko V.A., Yamashita S. (2007). Childhood thyroid cancer in Belarus, Russia, and Ukraine after Chernobyl and at present. Arq. Bras. Endocrinol. Metab..

[8] Fukushima Prefectural Oversight Committee Meeting for FHMS, Summary of the Results of Full-Scale Screening 2019. https://www.pref.fukushima.lg.jp/uploaded/attachment/336455.pdf.

